# 2017 Audited schedule of changes in net assets

**DOI:** 10.5195/jmla.2018.590

**Published:** 2018-10-01

**Authors:** Ray Naegele

**Affiliations:** Medical Library Association, 65 East Wacker Place, Suite 1900, Chicago, IL 60601-7246

The table below summarizes the association’s financial status as of December 31, 2017. For a more complete audit report and related information, see the Audited Financial Statements (members only). This report includes balance sheets, fund status reports, budgeted and actual revenues and expenditures, and a schedule of investments. Members may obtain a copy of the audit report from MLA headquarters.[Table t1-jmla-106-e29]

**Table 1 t1-jmla-106-e29:**
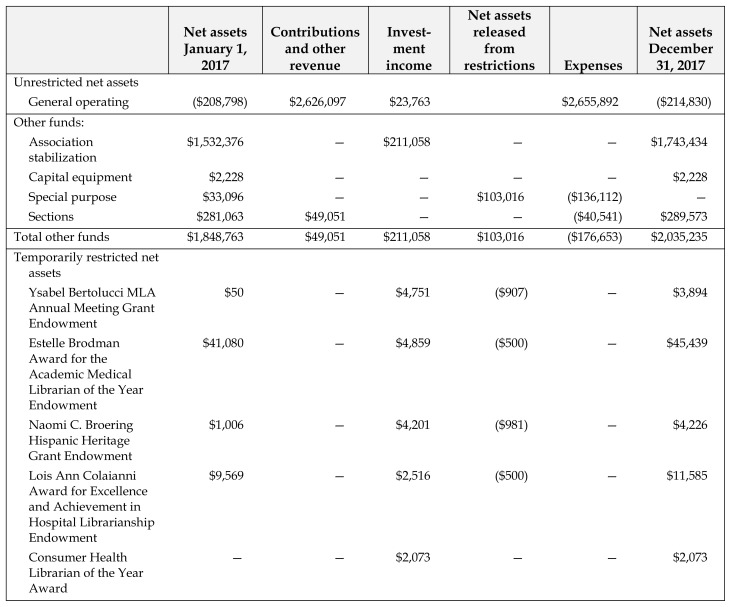

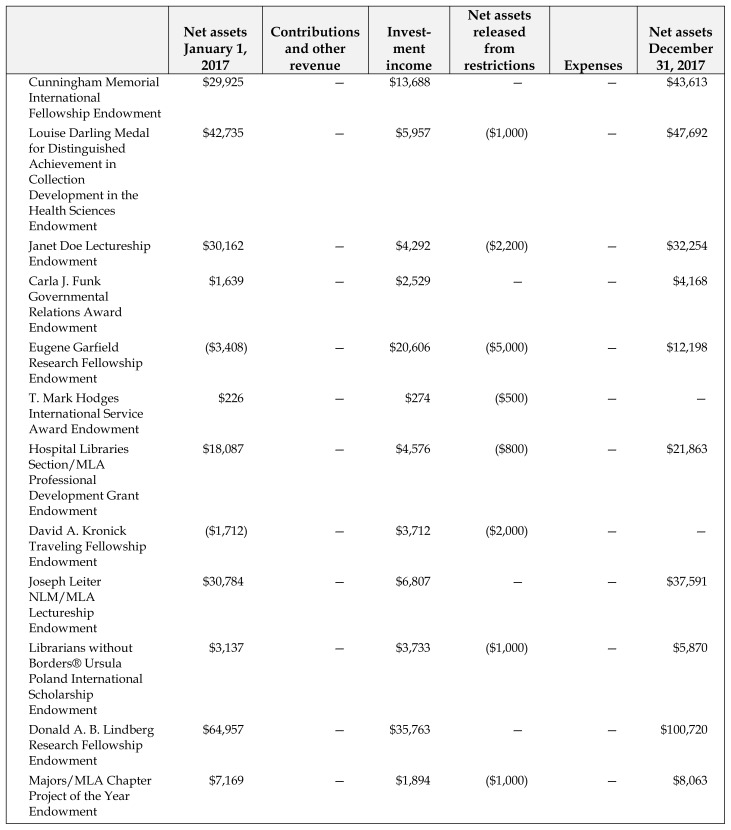

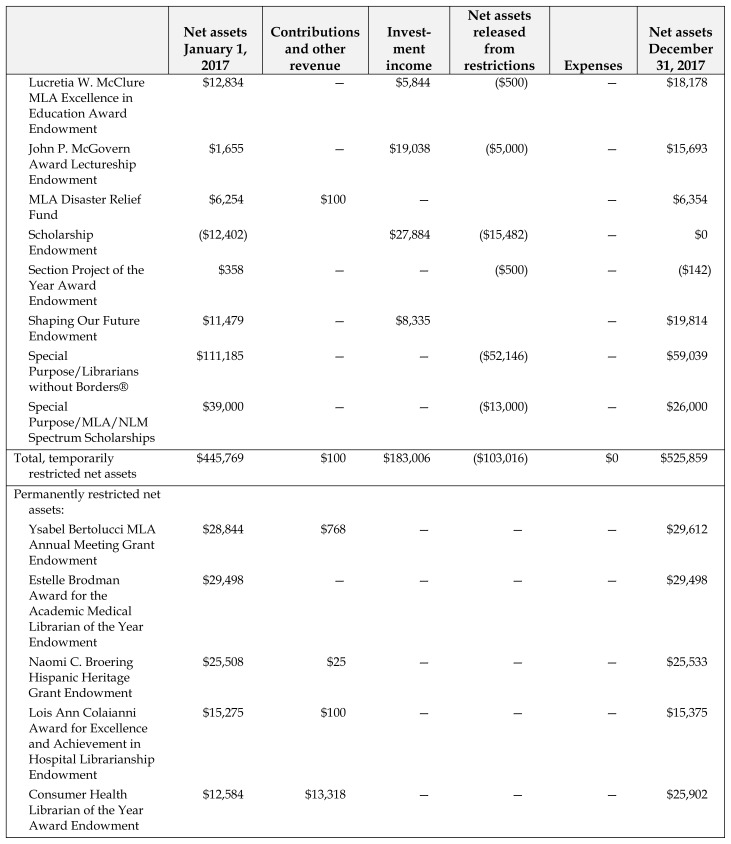

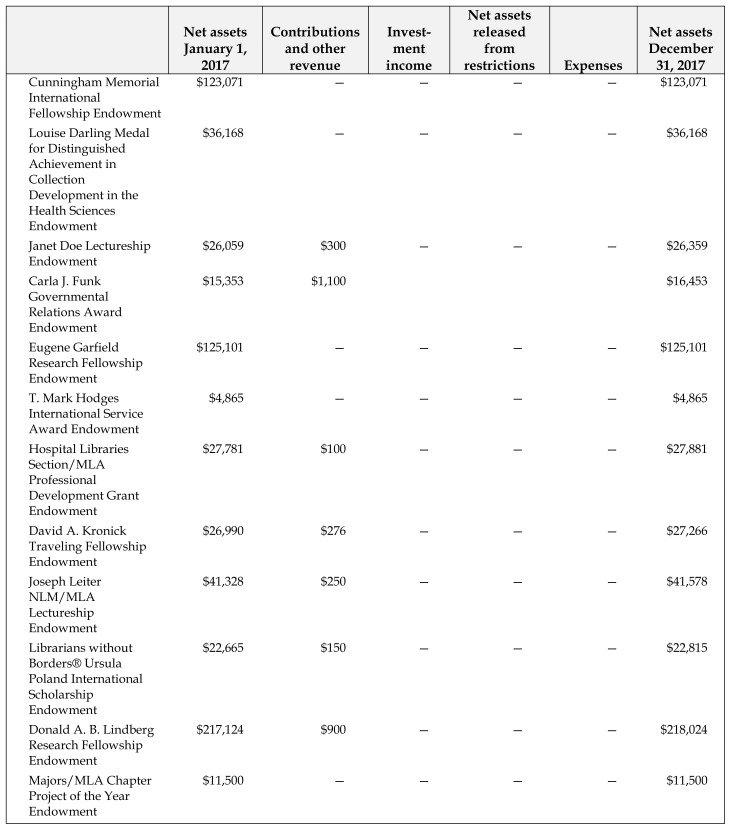
Medical Library Association schedule of changes in net assets by fund year ended December 31, 2017

	Net assets January 1, 2017	Contributions and other revenue	Investment income	Net assets released from restrictions	Expenses	Net assets December 31, 2017
Unrestricted net assets						
General operating	($208,798)	$2,626,097	$23,763		$2,655,892	($214,830)

Other funds:						
Association stabilization	$1,532,376	—	$211,058	—	—	$1,743,434
Capital equipment	$2,228	—	—	—	—	$2,228
Special purpose	$33,096	—	—	$103,016	($136,112)	—
Sections	$281,063	$49,051	—	—	($40,541)	$289,573

Total other funds	$1,848,763	$49,051	$211,058	$103,016	($176,653)	$2,035,235

Temporarily restricted net assets						
Ysabel Bertolucci MLA Annual Meeting Grant Endowment	$50	—	$4,751	($907)	—	$3,894
Estelle Brodman Award for the Academic Medical Librarian of the Year Endowment	$41,080	—	$4,859	($500)	—	$45,439
Naomi C. Broering Hispanic Heritage Grant Endowment	$1,006	—	$4,201	($981)	—	$4,226
Lois Ann Colaianni Award for Excellence and Achievement in Hospital Librarianship Endowment	$9,569	—	$2,516	($500)	—	$11,585
Consumer Health Librarian of the Year Award	—	—	$2,073	—	—	$2,073
Cunningham Memorial International Fellowship Endowment	$29,925	—	$13,688	—	—	$43,613
Louise Darling Medal for Distinguished Achievement in Collection Development in the Health Sciences Endowment	$42,735	—	$5,957	($1,000)	—	$47,692
Janet Doe Lectureship Endowment	$30,162	—	$4,292	($2,200)	—	$32,254
Carla J. Funk Governmental Relations Award Endowment	$1,639	—	$2,529	—	—	$4,168
Eugene Garfield Research Fellowship Endowment	($3,408)	—	$20,606	($5,000)	—	$12,198
T. Mark Hodges International Service Award Endowment	$226	—	$274	($500)	—	—
Hospital Libraries Section/MLA Professional Development Grant Endowment	$18,087	—	$4,576	($800)	—	$21,863
David A. Kronick Traveling Fellowship Endowment	($1,712)	—	$3,712	($2,000)	—	—
Joseph Leiter NLM/MLA Lectureship Endowment	$30,784	—	$6,807	—	—	$37,591
Librarians without Borders® Ursula Poland International Scholarship Endowment	$3,137	—	$3,733	($1,000)	—	$5,870
Donald A. B. Lindberg Research Fellowship Endowment	$64,957	—	$35,763	—	—	$100,720
Majors/MLA Chapter Project of the Year Endowment	$7,169	—	$1,894	($1,000)	—	$8,063
Lucretia W. McClure MLA Excellence in Education Award Endowment	$12,834	—	$5,844	($500)	—	$18,178
John P. McGovern Award Lectureship Endowment	$1,655	—	$19,038	($5,000)	—	$15,693
MLA Disaster Relief Fund	$6,254	$100	—		—	$6,354
Scholarship Endowment	($12,402)		$27,884	($15,482)	—	$0
Section Project of the Year Award Endowment	$358	—	—	($500)	—	($142)
Shaping Our Future Endowment	$11,479	—	$8,335		—	$19,814
Special Purpose/Librarians without Borders®	$111,185	—	—	($52,146)	—	$59,039
Special Purpose/MLA/NLM Spectrum Scholarships	$39,000	—	—	($13,000)	—	$26,000

Total, temporarily restricted net assets	$445,769	$100	$183,006	($103,016)	$0	$525,859

Permanently restricted net assets:						
Ysabel Bertolucci MLA Annual Meeting Grant Endowment	$28,844	$768	—	—	—	$29,612
Estelle Brodman Award for the Academic Medical Librarian of the Year Endowment	$29,498	—	—	—	—	$29,498
Naomi C. Broering Hispanic Heritage Grant Endowment	$25,508	$25	—	—	—	$25,533
Lois Ann Colaianni Award for Excellence and Achievement in Hospital Librarianship Endowment	$15,275	$100	—	—	—	$15,375
Consumer Health Librarian of the Year Award Endowment	$12,584	$13,318	—	—	—	$25,902
Cunningham Memorial International Fellowship Endowment	$123,071	—	—	—	—	$123,071
Louise Darling Medal for Distinguished Achievement in Collection Development in the Health Sciences Endowment	$36,168	—	—	—	—	$36,168
Janet Doe Lectureship Endowment	$26,059	$300	—	—	—	$26,359
Carla J. Funk Governmental Relations Award Endowment	$15,353	$1,100				$16,453
Eugene Garfield Research Fellowship Endowment	$125,101	—	—	—	—	$125,101
T. Mark Hodges International Service Award Endowment	$4,865	—	—	—	—	$4,865
Hospital Libraries Section/MLA Professional Development Grant Endowment	$27,781	$100	—	—	—	$27,881
David A. Kronick Traveling Fellowship Endowment	$26,990	$276	—	—	—	$27,266
Joseph Leiter NLM/MLA Lectureship Endowment	$41,328	$250	—	—	—	$41,578
Librarians without Borders® Ursula Poland International Scholarship Endowment	$22,665	$150	—	—	—	$22,815
Donald A. B. Lindberg Research Fellowship Endowment	$217,124	$900	—	—	—	$218,024
Majors/MLA Chapter Project of the Year Endowment	$11,500	—	—	—	—	$11,500
Lucretia W. McClure MLA Excellence in Education Award Endowment	$35,480	$1,309	—	—	—	$36,789
John P. McGovern Award Lectureship Endowment	$115,585	—	—	—	—	$115,585
Scholarship Endowment	$263,954	$38,959	—	—	—	$302,913
Shaping Our Future Endowment	$50,604	$1,400	—	—	—	$52,004

Total, permanently restricted net assets	$1,255,337	$58,955	$0	$0	$0	$1,314,292

Total all net assets	$3,341,071	$2,734,203	$417,827	$0	($2,832,545)	$3,660,556

